# An Ensemble Learning Approach for Electrocardiogram Sensor Based Human Emotion Recognition

**DOI:** 10.3390/s19204495

**Published:** 2019-10-16

**Authors:** Theekshana Dissanayake, Yasitha Rajapaksha, Roshan Ragel, Isuru Nawinne

**Affiliations:** Department of Computer Engineering, University of Peradeniya, Peradeniya 20400, Sri Lanka; yasitha.rajapaksha@eng.pdn.ac.lk (Y.R.); roshanr@eng.pdn.ac.lk (R.R.); isurunawinne@eng.pdn.ac.lk (I.N.)

**Keywords:** bio-signal processing, wearable computing, ensemble learning, electrocardiogram, machine learning

## Abstract

Recently, researchers in the area of biosensor based human emotion recognition have used different types of machine learning models for recognizing human emotions. However, most of them still lack the ability to recognize human emotions with higher classification accuracy incorporating a limited number of bio-sensors. In the domain of machine learning, ensemble learning methods have been successfully applied to solve different types of real-world machine learning problems which require improved classification accuracies. Emphasising on that, this research suggests an ensemble learning approach for developing a machine learning model that can recognize four major human emotions namely: anger; sadness; joy; and pleasure incorporating electrocardiogram (ECG) signals. As feature extraction methods, this analysis combines four ECG signal based techniques, namely: heart rate variability; empirical mode decomposition; with-in beat analysis; and frequency spectrum analysis. The first three feature extraction methods are well-known ECG based feature extraction techniques mentioned in the literature, and the fourth technique is a novel method proposed in this study. The machine learning procedure of this investigation evaluates the performance of a set of well-known ensemble learners for emotion classification and further improves the classification results using feature selection as a prior step to ensemble model training. Compared to the best performing single biosensor based model in the literature, the developed ensemble learner has the accuracy gain of 10.77%. Furthermore, the developed model outperforms most of the multiple biosensor based emotion recognition models with a significantly higher classification accuracy gain.

## 1. Introduction

Human–Computer Interaction (HCI) research is focused on making interaction with computers more productive and interactive. One of the methods used for improving the interaction between humans and computers is to provide emotional intelligence to computing systems. Such systems are capable of adapting depending on the emotional state of the user. Some examples for such systems include entertainment systems, healthcare systems, adaptive learning systems and computer games.

Previous studies have investigated different methods to provide emotional intelligence to computers. Among those methods, facial image based emotion recognition is the most widely used method since this method can recognize a wide range of emotion types [1,2]. Another approach is the speech signal analysis that determines the human emotion by analysing the patterns in the speech signal [3,4]. In addition, direct examination of the person is one of the most sophisticated ways for human emotion recognition [5,6]. These methods use different types of bio-signals to continuously monitor and detect human emotions. Due to its un-maskable nature, compared to facial emotion recognition and speech analysis, bio-signal based methods provide highly accurate results [7,8].

Several attempts have been made to use different types of bio-signals for emotion recognition. Some of the widely used bio-signals include the electrocardiogram (ECG), galvanic skin response (GSR), electromyogram and respiration. Some studies have combined multiple bio-signal devices to recognize emotions, whereas other studies have used a single biosensor such as ECG for capturing data for emotion recognition [8,9,10]. Despite the increased recognition accuracy, using multiple sensors might lead to user dissatisfaction. Compared to other biosensors, ECG is the most widely used biosensor because ECG signals are less noisy and they contain emotion related information [9,11].

Previous studies conducted to investigate the use of ECG signals have used different types of feature extraction methods. Some of those methods include heart rate variability (HRV), empirical mode decomposition (EMD), with-in beat analysis (WIB) and wavelet transforms [8,10]. Although those feature extraction methods are sophisticated methods, they suffer from low prediction accuracy. However, too little attention has been given to combining these sophisticated methods to achieve higher emotion recognition accuracy.

This study presents an ensemble learning approach for recognising human emotions using ECG signals. First, this investigation will discuss the protocol followed to obtain emotion-related ECG data through an experiment. Following that, this study will describe three broadly used ECG signal feature extraction methods, namely: HRV; EMD; and WIB analysis, and also a novel method proposed in this study based on the frequency spectrum of the ECG wave. Finally, this research will elaborate on the machine learning process that followed to create a classification model by combining the mentioned feature extraction methods. Briefly, the machine learning model takes a 20s window of an ECG signal and then classifies the signal to four distinct emotion classes, namely: anger; sadness; joy; and pleasure. The machine learning process first evaluates the capability of different ensemble learners for emotion classification. After analysing, as an additional step, a feature selection process is employed to improve the classification accuracy of each ensemble learner. This step is based on previous related studies on ensemble learners [12,13,14,15,16] and feature selection strategies for ensemble learners [17,18,19,20]. Finally, this research presents the selected features from the feature selection process and compares the obtained results with different ECG based emotion recognition models in the literature.

## 2. Related Work

In recent years, there has been an increasing amount of literature on human–computer interaction methods to provide emotional intelligence to computers. Emotional intelligence is widely used to develop emotionally-aware healthcare monitoring systems, computer games and entertainment systems and safe driving systems. In computer games, emotional intelligence can be used to evaluate the player’s affective state for dynamic game content generation [5,21,22]. Similarly, in vehicle safety systems, emotion recognition models are used to monitor the affective state of the driver while operating [23,24]. Furthermore, in health care systems, emotional intelligence is employed to monitor the emotional state of patients [6,25,26].

Rattanyu and Mizukawa [9] discuss speech analysis, facial feature analysis and bio-signal processing as the primary methods for emotion recognition. Firstly, speech based emotion recognition methods determine the emotion by analysing a given speech signal. The main drawback of this method is that the user needs to speak continuously if the system wants to figure out the emotional state. Secondly, facial image based recognition is another widely used method for emotion recognition [1,2]. Although it provides accurate predictions for emotions, the main problem in this method is that some people tend to mask their emotional states (social masking) while predicting [7,8]. Finally, bio-signal processing methods use different types of bio-signals to predict emotions. A bio-signal based method is an adequate solution for recognizing emotions compared to other methods. Because of its unmasked nature, bio-signals improve the predicting accuracy compared to facial image based recognition methods [8]. In addition, since it is available continuously, unlike speech based systems [3,4], the system can continuously identify the emotion level.

Numerous studies have attempted to use different types of bio-signals for detecting emotions [9,10,27]. Kim and André [8] developed an emotion recognition model incorporating four different biosensors: electrocardiogram, skin conductivity, electromyogram and respiration. In their investigation, they have achieved 70% accuracy for a person independent of emotion classification. The developed model was able to classify a given set of bio-signal patterns to four emotion classes: anger, sadness, pleasure and joy. In another major study, Rattanyu and Mizukawa [9] developed an emotion recognition model using ECG signals with a classification accuracy of 61%. In a study that set out to develop a neural network based emotion recognition model, Yoo et al. [28] developed a model incorporating ECG signals and skin conductivity. In their study, they have formed a classification model with 80.1% accuracy. In another major investigation, Ayata et al. [29] developed an emotion recognition model to classify arousal and valence using galvanic skin response. The mentioned model incorporates features from empirical mode decomposition and statistical analysis methods. Among multi-sensor based emotion recognition models, the model developed by Nazos et al. [30] has the ability to recognize sadness, anger, surprise, fear, frustration and amusement with up to 83% accuracy. Furthermore, the recent investigation by Gouizi et al. [31] has an accuracy of 83% for recognizing six emotions by using six different biosensors. More information on biosensor based emotion recognition can be found in the recent state-of-art reviews by Jeritta et al. [32] and Egger et al. [33].

Murugappan et al. [34] discuss the challenges and limitations in multi-sensor based emotion recognition. One of the major challenges is the increased computational complexity due to multiple sensor data streams and algorithmic requirements. The other factor is the limitation to subjects’ freedom (movements) due to multiple sensor probes, wires, etc. By building on the concept of simplicity, they have been able to develop an emotion recognition model with a classification accuracy of 66.48% by only using ECG signals. Considering all of these factors, the selected method should be able to provide high emotion recognition accuracy with a minimum number of sensors.

A number of studies have examined the use of ECG signals for emotion recognition [7,10,27,34,35]. An ECG based method is an adequate solution due to four important reasons. Firstly, the ECG signal is a result of activities in the heart that has nerve endings from the autonomic nervous system that governs the behaviour of each emotion [11]. Secondly, ECG sensors can be used as a wearable device [36]. Thirdly, it is convenient to use because ECG signals can be captured from different parts of the body [37]. Finally, it has a high amplitude compared to other biosensors [9].

To date, various methods have been developed and introduced to extract features from ECG signals. One commonly used method is the heart rate variability analysis [8,28,38]. HRV analysis is a broadly used method in biomedical engineering applications [39]. The method developed by Ferdinando et al. [38] using HRV analysis had an accuracy of around 59% for identifying arousal and valence state of a person. Similarly, another study that developed an emotion recognition model incorporating ECG signals and skin resistance had 80% accuracy for recognizing the four quadrants of the discrete emotional model [40].

Another widely used method is the empirical mode decomposition. EMD is one of the well-structured approaches to analysing non-stationary and nonlinear data [41]. Furthermore, according to investigations done by Manjula and Sarma [42], compared to wavelets, EMD performs better while extracting spectral power based features. Foteini et al. [43] point out that the first six intrinsic mode functions generated from the EMD method relates to a specific activity in the heart. Developing on that, the number of studies have used empirical mode decomposition for analysing ECG signals [7,27]. Jerrita et al. [7] investigated the use of Hilbert–Huang transform (HHT) for EMD based feature extraction and came up with a classification model with 54% accuracy for identifying six emotions in the discrete emotion model [40].

With-in beat analysis is another method for ECG based emotion recognition that has a high emotion recognition accuracy compared to EMD and HRV methods. This method was introduced by Rattanyu and Mizukawa [9] for recognising six emotions in the emotional spectrum. Their model was able to identify an emotion with up to 61% accuracy using ECG signals.

Some of the studies have used discrete Fourier transform (DFT) to extract frequency domain features from the ECG signal. Jerritta et al. [10] discuss the advantages of using frequency domain features compared to EMD based features. They claim that, unlike EMD features that provide an idea about the local properties of the ECG wave, the DFT method provides information about the frequency content of the signal. In their study, they have achieved 54% accuracy for recognizing neutral, happiness, sadness, fear, surprise and disgust emotions from ECG signals utilizing DFT based features of ten intrinsic mode functions derived from the EMD method.

Collectively, most of the studies have used different analysis methods to extract features from ECG signals. In HRV analysis, the HRV time series is generated only by considering the R–R interval variations of the ECG wave. However, the features extracted from this method represent features from both the time domain and the frequency domain of the HRV wave. Similarly, the EMD technique decomposes the signal into a set of oscillating signals. The features extracted from this method also correspond to a set of fragmented features that has correlations to an ECG wave. However, compared to these two methods, the within beat method analyses the raw ECG wave in the time domain. In addition, compared to frequency domain based features extracted by EMD and HRV methods, the DFT method provides an overview of the frequency domain of the raw ECG wave. Each of these approaches has its own advantages, and the features generated by all of these techniques represent a broad range of features that correspond to different domains and spaces of the ECG wave. However, most of the ECG based feature extraction methods in the literature have emotion recognition capability around 55% for different types of classification requirements.

Together, these studies highlight the need for an accurate emotion recognition model with a minimum number of biosensors. The studies presented thus far provide evidence that ECG is the best method for capturing bio-signals because ECG signals contain emotion-related information. In addition, considering the accuracies gained from the classification models, there is a need for higher classification accuracy. However, the methods represented in the literature extract a wide range of features from the ECG wave, and they are sophisticated methods for examining time-varying data. Up to now, a number of studies have investigated different approaches for ECG based emotion recognition. However, up to now, no one has investigated the feasibility of combining well-known ECG based feature extraction methods to select an optimal set of features that gives higher emotion classification accuracy.

Considering most of the studies mentioned in the literature, it is apparent that the majority of them have used traditional single learner algorithms as the prediction model. Most of the considered algorithms include support vector machines, K-nearest neighbour, Fisher analysis and artificial neural networks. Even though most of the mentioned algorithms are well-equipped techniques, a majority of them lack the ability to recognize emotions with a higher classification accuracy. Recently, ensemble learning methods have been used to improve the classification accuracy of various problems in different domains, and they have gained significant accuracy improvements after applying these techniques [15,16,19]. Furthermore, even the research in the domain of biomedical signal analysis have also used these ensemble techniques to improve the model performance [12,17,18].

Even though there has been an extensive amount of research conducted in defining primal emotions for humans, yet, while developing prediction models, research has selected different emotions as their target emotions [44]. This investigation is based on the 2D emotional model proposed by J. A Russel [45] where the emotions are placed in a 2D arousal and valance space. To be more broad in the aspect the emotion selection, this study considers the primal emotion of each emotional quadrant as the selected emotion. This selection will improve the diverse nature of the predictions made in the study. Furthermore, there are similar studies that had the same set of emotions as their targets, and those gained classification accuracies will be beneficial while benchmarking the developed model. Therefore, the analysis of this study is focused on recognizing four primal emotions, namely: anger; sadness; pleasure; and joy. Additionally, this study presents the classification results of two models developed incorporating two additional emotions in the emotional spectrum. Furthermore, a complete overview of the emotions and their organization in the arousal valance space is described in the next Section 3.1.3.

The main objective of this paper is to evaluate the capability of ensemble learners for biosensor based human emotion recognition that requires higher prediction accuracies. This research combines four ECG based feature extraction methods, namely: HRV; EMD; WIB; and DFT based. The first two techniques are the most widely used methods in the literature, and this study uses the with-in beat method because of its high emotion recognition accuracy. Additionally, this study introduces a novel method that extracts a set of frequency-domain features from ten frequency bands of the ECG wave employing discrete Fourier transform (named as TFB features). As an additional step for the ensemble learning procedure, the machine learning analysis of this paper selects a set of optimal features by combining the mentioned feature extraction methods for recognising anger, sadness, joy and pleasure.

## 3. Material and Methods

As discussed earlier, the principal objective of this research is to suggest an ensemble learning approach for ECG based emotion recognition by combining four ECG feature extraction methods. The selected feature extraction methods can be listed as follows: HRV, EMD, WIB and TFB. Section 3.1 of the methodology describes the 2D emotion model and ECG signal acquisition. Moving forward, Section 3.2 addresses the signal pre-processing algorithms. After that, Section 3.3 describes selected feature extraction methods in detail. Finally, Section 3.4 of the methodology illustrates the machine learning process.

### 3.1. Experiment for ECG Data Collection

This section describes the process conducted to acquire data from subjects to develop a machine learning model. The first section of this subsection provides insight into selecting a suitable ECG sensor for capturing ECG data. The second section explains the ECG data capturing algorithm developed. Moving forward, the following section talks about the adapted discrete emotional model. The last section explains the ECG data collecting experiment in detail.

#### 3.1.1. ECG Sensor

**Figure 1:**
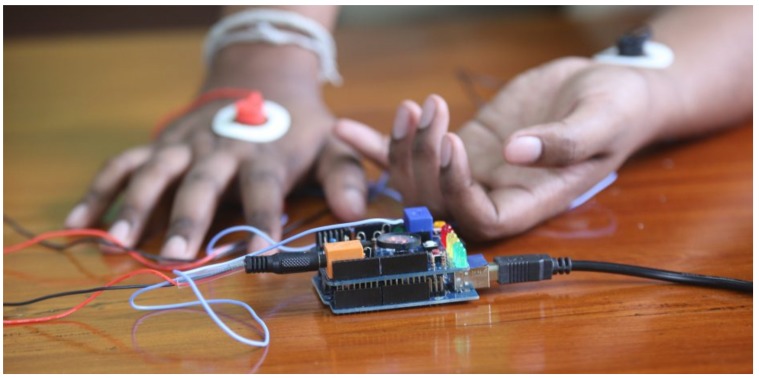
SpikerShield Heart and Brain sensor.

As there are various types of hardware out there that records ECG signals, hardware selection was done based on a few factors. Firstly, the subject should not feel restricted while wearing the hardware as it has a direct impact on the comfort of the subject. Secondly, recorded ECG signals should not be too noisy as too much noise will make the processing difficult. Finally, the hardware should be financially affordable. After considering all, a Spiker-Shield Heart and Brain sensor was selected as it is affordable and has an inbuilt noise sensor. The subject only needs to have a few plasters on their hand in order to record the ECG signals, which makes it not too disturbing to the subject as well. Figure 1 shows an image of the wearable sensor used.

#### 3.1.2. Signal Collection

A simple algorithm was developed to acquire signals from subjects by communicating with an Arduino microcontroller. The sampling rate of the signal was set to 1000 Hz, and the baud rate of the serial communication unit was adjusted to 115,200 bps. Since the emotion-related changes can be observed in 3–15s of the ECG signal frame, the length of the ECG signal was set to a 20 s interval [32]. The algorithms sent the captured 20 s ECG wave frame to the data collection node via a Nodejs asynchronous function interface. Then, the data collection node captures the data and writes the values to a data file (.txt) indicating the subject ID, captured time and the emotion. Later, this information is used to filter the emotion elicited data frame from the data space.

#### 3.1.3. Discrete Emotional Model

**Figure 2:**
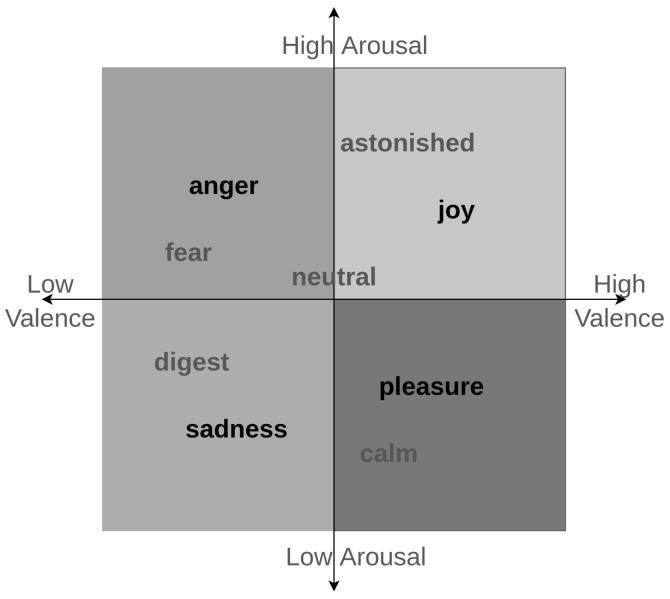
Discrete emotional model.

As shown in Figure 2, the discrete 2D emotional model [45] places all human emotions on two axes, namely: arousal; and valence. The first quadrant of the emotional model includes highly aroused and valenced emotions. This quadrant holds joy as its primal emotion and other sub-emotions such as excited, astonished, and delighted as secondary emotions. The second quadrant of the emotional model, which represents low aroused and high valenced emotions, includes emotions like pleasure and calm. Next, the third quadrant of the emotional model incorporates emotions such as sadness and digest. Finally, the fourth quadrant of the emotion model includes anger, fear and annoyed emotions, which represents the low valenced high aroused scenarios.

#### 3.1.4. Experiment

The designed experiment captures six emotions in the discrete emotional model, namely: joy; sadness; pleasure; anger; fear; and neutral. A majority of these emotions represent the primal emotions of the emotion spectrum, and those are the emotions that were intended be identified in this study using ECG signals. The other emotions (fear and neutral) were chosen to conduct the comparative analysis with the literature.

As for the design of the experiment, firstly, a set of videos were collected by consulting with domain experts to elicit selected target emotions. Each of these videos was 3–10 minutes in length. Subjects were invited into a disturbance-free environment and sensors were fixed on to them in order to record ECG signals. Then, each and every video was shown to the subjects having two minutes of breaks in between. At the end of each video, subjects were asked to write down their emotional experience throughout the video in a pre-designed feedback paper, highlighting points of emotional climaxes. If the subject’s emotional climaxes match with the target emotion, then it is marked as a successful attempt (i.e., a hit—see the Table 1). These climaxes were synchronized with the ECG data collection unit, and later this information was used to filter the emotion-related ECG signals. This is achieved by measuring the ECG signals with time information, and then by matching the time of the emotional climax and the signal time.

Table 1 above illustrates the information regarding the selected video clips and durations. Even though they have marked as a specific emotion-related video, subjects were not aware of the intended emotion level of each. Furthermore, to further eliminate the bias in the results, the order of the video play was also changed. Collectively, compared to other three primal emotions, anger video has the lowest hit rate, and this phenomenon was also observed in previous studies [46]. Despite that, other selected videos had a significantly better chance of eliciting target emotions.

**Figure 3:**
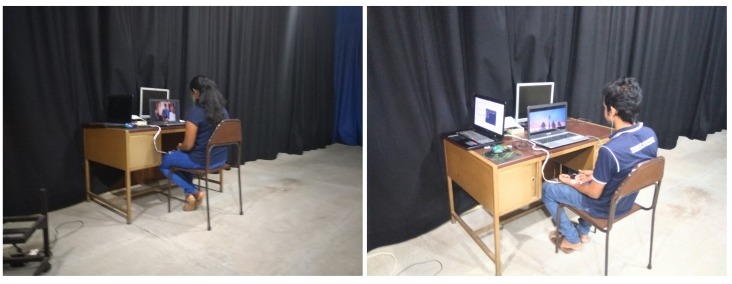
Experiment environment.

Figure 3 shows the experimental setup and the environment while two subjects were going through the designed experiment. Furthermore, subjects were of both genders, between 22–26 years of age and a total number of 25 subjects participated in the experiment. Out of these 25, ECG signals of three participants had to be removed due to noise issues and signal anomalies. The rest of the ECG signals were used for the proceeding work.

The final filtered data set contains 488 20 s ECG waves that include, 105 anger waves, 110 sadness waves, 174 joy waves and 99 pleasure waves. Furthermore, it comprises 165 data frames of fear emotion and 103 from neutral emotion.

### 3.2. Signal Pre-Processing

The pre-processing procedure of the signal consists of three main steps: filtering, de-trending and smoothing. First, a Butterworth bandpass filter with a frequency range of 0.05–100 Hz was used to remove the noise from the ECG signal [8]. The resulting signal shows a trended pattern as a result of filtering near the DC component. Therefore, Algorithm 1 was used to stabilize the signal.

**Algorithm 1** De-Trending
**Require:** x[n]|n∈{0,1,⋯,N-1}
**Require:** 0≤K≤N(default8)
  
----Splitthesignalx[n]intoKsegments.
  
K←8
  
----DefineX[K]size(K,N/K)
  
X[K]=split(x[n],8){∀k∈[1,2,⋯,K]}
  
----Foreachsegmentcompute
  
**for** $x_{k}\left[n\right]\in X\left[K\right]$ **do**
     
$----Fit\;the\;k^{th}\;segment\;to\;a\;2^{nd}\;order\;polynomial$
     
$poly\_coeff_{k}=polyfit\left(x_{k}\left[n\right],degree=2\right)$
     
----Predictthetrendusingpolynomial
     
$trend_{k}=poly\_coeff_{k}\left[t\right]$
     
----Removethetrend
     **for** t∈[0,⋯,N/K] **do**        
$x_{k}\left[t\right]\leftarrow x_{k}\left[t\right]-trend_{k}\left[t\right]$
     
**end for**
  
**end for**
  
----Concatenateallsegments
  
ECG[n]=concatenate(X[K])
  
**return** ECG[n]



**Figure 4:**
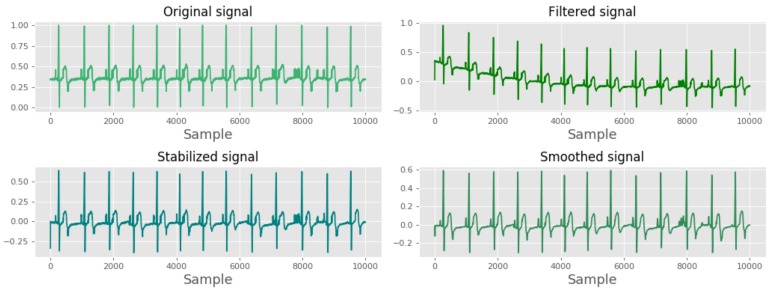
Pre-processing algorithm results.

Algorithm 1 describes the de-trending procedure used to stabilize the signal. The algorithm takes a signal x[n] with *N* samples and stabilizes the signal by dividing the signal into small segments (*K* number of segments). First, the algorithm fits each signal segment to a second order polynomial. After that, the trend of the segment is estimated by providing the time variation of the signal. Then, the predicted trend, which is a time variation of a second order polynomial, is reduced from the original signal. After de-trending, the resulting signal was further smoothed using a Gaussian kernel, and it was made sure that the smoothing procedure preserves the vital information of the wave. Figure 4 illustrates the resulting signals after conducting each step.

### 3.3. Feature Extraction Methods

This subsection of the study discusses the selected feature extraction methods in detail. The first section of this subsection explains the PQRST detection algorithm. Then, the second section describes the process of generating the HRV time series and the HRV analysis that is based on the generated HRV time series. Moving on, the next section of the feature extraction methods talks about empirical mode decomposition based features. After that, the third section discusses the with-in beat analysis technique. Finally, the last section of this subsection presents the novel frequency band based feature extraction technique used for ECG based feature extraction.

#### 3.3.1. PQRST Detection

**Figure 5:**
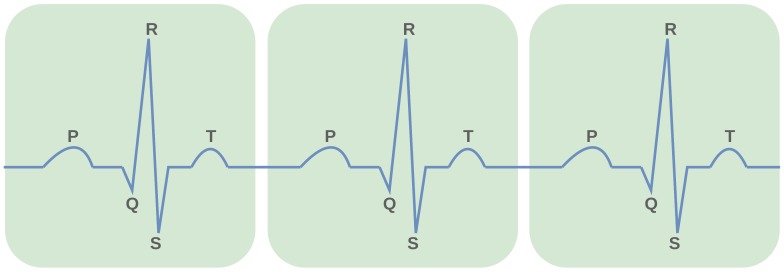
PQRST wave locations.

As Figure 5 shows, the ECG signal pattern is a result of a series of waves associated with the activities of the heart [47]. The ECG pattern consists of the P wave, QRS complex followed by that the T wave. Each of these waves corresponds to a specific activity in the heart (repolarization or depolarization).

**Figure 6:**
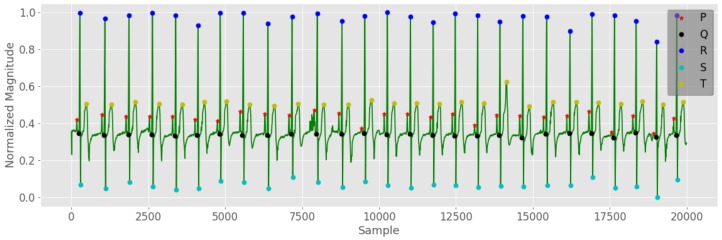
PQRST detection algorithm results.

To detect PQRST wave positions in an ECG signal, first, a simple algorithm was designed to find the R peak locations of each QRS complex. Then, the identified R peak locations were used to segment out QRS complexes from the ECG signal. After that, since the ECG signal has a specific pattern, local minima and local maxima detection methods were employed to figure out PQST wave locations from each segmented QRS complex. Figure 6 illustrates the PQRST positions discovered using a PQRST detection algorithm. These computed locations were later employed in HRV analysis and WIB analysis. In HRV analysis, only R peak locations are used to compute a set of diverse features, whereas all statistical features of WIB analysis are computed considering different peak-to-peak intervals (PR, RS, QRS, etc.).

#### 3.3.2. Heart Rate Variability Analysis

**Figure 7:**
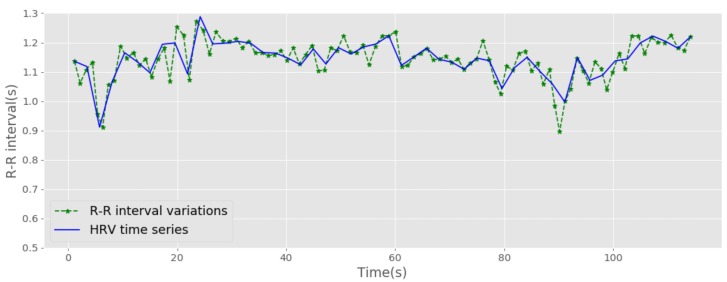
Heart Rate Variability (HRV) time series.

Heart rate variability analysis (HRV) is one of the most commonly used methods for ECG feature extraction [8,38]. To compute the HRV time series, first, the ECG signal was processed using the PQRST position detection algorithm. After that, the detected R positions (R peaks) were used to compute the R–R interval variations of the ECG wave. Finally, the R–R intervals and the corresponding cumulative sums of R–R intervals were used to compute the interpolated HRV time series. Figure 7 shows a computed HRV time series for a selected subject.

The HRV analysis method can be treated under three headings: time domain analysis, frequency domain analysis and geometric methods based analysis. Firstly, time domain analysis relates to a set of statistical features and heart rate variability specific features extracted from HRV time series. Secondly, the frequency domain analysis refers to a collection of features extracted through high frequency **HF** (0.15 to 0.4 Hz), low frequency **LF** (0.04 to 0.15 Hz) and very low frequency **VLF** (0.0033 to 0.04 Hz) bands of HRV time series. Thirdly, geometric based analysis associates with a set of features obtained from Poincaré geometric plots. Table 2 presents an overview of extracted features and their respective domains.

The standard deviation of NN intervals (sdnn), the mean value of NN intervals (m_nn), root mean square of NN intervals (rmssd) and max value of NN (m_nn) intervals are taken as statistical features. The number of pairs of neighbouring NN intervals varying by more than 50 ms (nn50) and the NN50 over the total number of NN intervals (pNN50) are extracted as additional time-domain features. Here, NN refers to the Normal-to-Normal interval, and it can be also seen as the R–R interval that was adopted in this study. Spectral powers of LF (lf), VLF (vlf) and HF (hf), power ratios of LF/HF (lf_hf), the low-frequency power in normalized units (lfnu) and the total power (total_power) are used as frequency-domain features. The Poincaré plots transfer the R–R intervals to a different geometric domain and sd1 and sd2 features are calculated as the geometric deviations between consecutive R–R intervals. A detailed explanation of each method and respective feature notations can be found in [8].

#### 3.3.3. With-in Beat Features of the ECG Signal

This section implements the method proposed by Rattanyu and Mizukawa [9] for emotion recognition from ECG signals using with-in beat features. With-in beat information of ECG signal includes PR interval, ST interval and QRS interval (i.e., QS). Unlike the HRV method, this method considers the variation of inner pulses of the ECG signal. The with-in beat method computes five different statistical features from each interval. They are mean (*mean*), maximum (*max*), minimum (*min*), median (*median*) and standard deviation (*sd*).

To compute with-in beat features, first, the ECG signal was sent to the PQRST detection algorithm. The algorithm returns the locations of identified PQRST positions, and those locations are then used to compute the considered intervals. Table 3 illustrates the computed features and their corresponding notations. Each feature corresponds to an interval (IN) is in the form of Label (1):

(1) IN=[min_IN,max_IN,sd_IN,mean_IN,median_IN].

#### 3.3.4. Empirical Mode Decomposition Based Features

**Figure 8:**
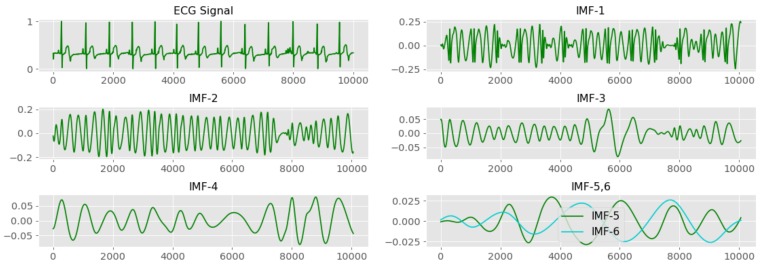
The first six IMFs and the base ECG wave.

Empirical mode decomposition decomposes a given signal to a finite number of signals called intrinsic mode functions (IMF). This study computes four different features from each IMF. They are spectral power of IMF in the time domain, the spectral power of IMF in the frequency domain, instantaneous frequency of IMF and spectral power of the instantaneous frequency spectrum of the IMF. Collectively, this EMD based feature extraction method extracts 24 features from ECG signal in both time domain and frequency domain. Figure 8 illustrates the first six IMFs generated from the EMD procedure with the original ECG wave.

Spectral power in the time domain (spec_p) of each IMF was estimated using the following Label (2). (In this equation, x[i] refers to a discrete signal with N samples.)

(2) $$\begin{array}{c} Power=\frac{1}{N}\sum_{i=1}^{i=N}x{\left[i\right]}^{2}. \end{array}$$

The Welch’s [48] method was used to compute the spectral power in the frequency domain (spec_pf), and Hilbert transform was adopted to estimate the instantaneous frequency (mean_if) of the IMF. The spectral power of the instantaneous frequency spectrum (spec_pf) was calculated using (2). The feature vector computed using $IMF^{i}$(i∈[1,2,⋯,6]) is in the form of Label (3),

(3) $$\begin{array}{c} feature_{i}=\left[\begin{array}{cc} spec\_p_{i} & spec\_pf_{i}\\ mean\_if_{i} & ins\_p_{i} \end{array}\right]. \end{array}$$

#### 3.3.5. Ten Frequency Band Analysis

**Figure 9:**
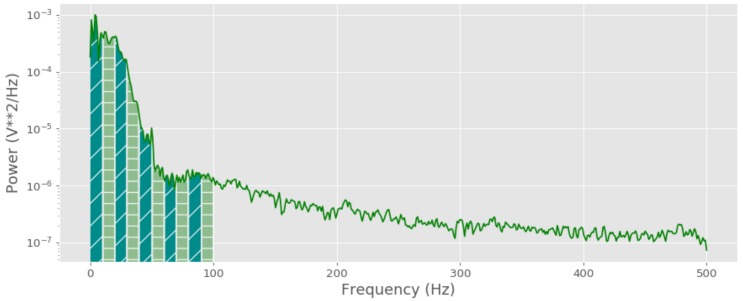
Spectral power variation.

A number of studies have explored the use of different frequency band based features for emotion classification [8,34]. Elaborating on that, this subsection of the study presents ten frequency band analysis (TFB) for emotion recognition. As shown in Figure 9, the frequency range of the ECG signal falls between 0–100 Hz. The developed method divides the ECG frequency range into ten different sub-bands having 10 Hz bandwidth for each and computes the spectral power of each sub-band. This analogy will provide a different set of frequency domain features compared to HRV time series based features and EMD based features.

The selection process of ten frequency bands was based on an empirical study on different spectral power bands within the range of 0–100 Hz. This selection criterion does not consider the physiological aspects of each frequency band, and even the selection of spectral power, as the measure is based on similar related studies that used spectral power as one of the signal features. First, a smaller analysis was conducted to find the optimal sub-band value varying from 1–20 Hz, while making sure the collected number of bands have an adequate number of feature values for classification. Then, the final result, which showed the highest ensemble based classification accuracy, was chosen as the number of bands (in this case, 10 bands at 20 Hz each).

First, Welch’s method was used to compute the frequency power spectrum of the ECG signal. This method computes the frequency power spectrum by splitting the signal into a set of overlapping segments and taking the average squared magnitude of each frequency component ($f\;\in [0,\cdots ,f_{s}/2]$).

The Welch’s method takes a discrete signal with N samples (x[n]), sampling frequency of the signal ($f_{s}$), length of a segment ($l_{seg}$), overlapping length of a segment ($l_{over}$) and the window function (w[n]) as input arguments, and then returns the power spectrum ($P_{x}$) and the frequency distribution ($F_{x}$) of the given signal. Algorithm 2 describes an overview of the steps followed in Welch’s method.

**Algorithm 2** Welch’s Method
**Require:** x[n]|n∈{0,1,⋯,N-1}
**Require:** $0\leq l_{seg}\leq N\;|\;\in N$
**Require:** $0\leq \;l_{over}\leq N\;|\;\in N$
  
----Splitthesignalx[n]intoKsegments.
  
$----X\left[K\right]\;matrix\;for\;all\;segments\;(K\times \left(l_{seg}\right))$
  
X[K]←[][]
  
$K\leftarrow N/l_{seg}$
  
$X\left[K\right]=split\left(x\left[n\right],\;l_{seg},\;l_{over}\right)\;\;\left\{\;\forall k\in \left[1,\cdots ,K\right]\right\}$
  
$----Convolve\;each\;segment\;x_{k}\left[t\right]\;with\;w\left[n\right]$
  
$----Compute\;the\;FFT\;of\;x_{k}\left[n\right]$
  
$----F\left(j\omega \right)\;matrix\;for\;frequency\;power\;(K\times \left(l_{seg}/2\right))$
  
F(jω)←[][]
  
**for** $x_{k}\left[n\right]\in X\left[K\right]$ **do**
     
$\left(x_{k}*w\right)\left[n\right]=convolve\left(x_{k}\left[n\right],w\left[n\right]\right)$
     
$F\left(j\omega_{k}\right)=FFT\left(x_{k}\left[n\right]\right)$
  
**end for**
  
----Computefrequencyvalues
  
$F_{x}=0:\left(f_{s}/2\right)\;\times \;1/\left(l_{seg}/2\right):f_{s}/2$
  
----Foreachfrequencycomputesquaredmagnitude
  
$P_{x}\left[f\right]\leftarrow \left[\;\right]$
  
**for** $f\in [0,\cdots ,f_{s}/2]$ **do**
     
**for** $f_{k}\left[i\right]\in F\left(jw\right)$ **do**
       
$P_{x}\left[f\right]+=f_{k}{\left[f\right]}^{2}$
     
**end for**
  
**end for**
  
----Computeaveragevalues
  
**for** $p_{f}\in P_{x}\left[f\right]$ **do**
    
$P_{x}\left[f\right]=p_{f}/K$
  
**end for**
  
**return** $P_{x}\left[f\right]$



A more optimized version of the Welch’s method in Scipy API [49] was used to compute the frequency power spectrum of the ECG signal by setting $l_{seg}$ as 256, $l_{over}$ as 128 and w[n] as the Hanning window.

After computing the frequency power spectrum of the ECG wave, a 0–100 Hz frequency range of the ECG frequency spectrum was filtered out and divided into ten sub-bands. Then, the trapezoidal integration was used to compute the spectral power of each sub-band. The derived ten spectral power features can be expressed as following Label (4):

(4) $$\begin{array}{c} features=\{band_{i}|\;i\in \left[1,2,\cdots ,10\right]\}. \end{array}$$

### 3.4. Emotion Recognition Model

This section describes the machine learning process that is used to develop a machine learning model for identifying four major emotions: anger, sadness, pleasure, joy. The pre-processed data contains 488 data frames and 63 features for each data frame. The machine learning procedure of this study is divided into two parts: the (1) Ensemble learner based machine learning process, and the (2) Ensemble learner and feature selection based machine learning process.

The first strategy of this analysis is based on the traditional way of ensemble learning where the ensemble learner chose the features and dynamically derives a set of diverse learners. The second technique is inspired by recent studies related to feature selection before ensemble learning [13,14,19]. Both of the strategies mentioned employ six popular ensemble learning methods that cover most of the modern bagging and boosting techniques. Furthermore, adopted feature selection methods represent a set of diverse techniques for machine learning feature selection (statistical, search based and algorithmic).

**Adopted Ensemble learners**

- Random Forest Classifier
- Extra Tree Classifier
- Gradient Boost Classifier
- ADABoost Classifier with Support Vector Machine (SVM)
- ADABoost Classifier with Decision Tree
- ADABoost Classifier with Naive Bayes

**Adopted feature selection methods**

- Recursive Feature Elimination
- Chi-Square Test
- P Test
- Feature Selection by Random Forest Classifier
- Feature Selection by Extra Tree Classifier
- Feature Selection by Random SVM

Each of these procedures is followed by a model parameter optimization process and a model evaluation process. The Grid Search algorithm [50] was used as the model parameter optimizer and traditional 10-Fold Cross-validation was used to evaluate the model. Prior to the machine learning process, the data was normalized by a Robust scalar, and all of the algorithms used in this section were taken from Python Scikit API [50].

## 4. Results and Discussion

The results section of this paper will elaborate on four Sections. Section 4.1 results from ensemble methods without feature selection, Section 4.2 results from ensemble methods with feature selection, Section 4.3 results overview and Section 4.4 computational requirement analysis.

Section 4.4 computational requirement analysis for combined features. As explained, the first two sections of the results and discussion will present the data gathered from ensemble learning and prior feature selection. Furthermore, this section will investigate whether feature selection is a worthy step for ensemble learning algorithms. Moving on, the results overview section compares the final results with different classification models in the literature by discussing emotion elicitation methods, experimental procedures and limitations. The final sector describes the computational requirements of each adopted feature extraction method, and then provides reasons for selecting combined analysis with ensemble learning as an optimal method for ECG based emotion recognition. It should be noted that the results mentioned in this section is for recognizing four major emotions in the 2D emotional model.

### 4.1. Ensemble Learning

Table 4 illustrates the results obtained for different ensemble learning techniques while presenting model parameter optimization results obtained using the Grid Search Algorithm. According to the results, an Extra Tree Classifier shows the highest prediction capability, which is 70.09% with a standard deviation of 3.34%. Furthermore, the ensemble model developed using the Random Forest classifier has the second-highest classification accuracy, and this value is slightly lower than the capability of an Extra Tree Classifier. Examining other techniques, the Gradient Boost classifier also shows adequate performance for emotion classification. However, the ADABoost ensemble with different base learners shows relatively lower prediction accuracies.

### 4.2. Ensemble Learning with Feature Selection

Table 5 illustrates the classification accuracies after selecting features employing different feature selection techniques. This table only presents the accuracies gained from the three best performing models observed in the previous section. In general, different models show different classification performances while undergoing diverse feature selection methods. For instance, Random Forest Classifier shows the highest accuracy for Model based feature selection, whereas a Gradient Boost classifier provides better results for the Recursive Feature Elimination technique. Examining all results, it is apparent that the model selection procedure improved the individual accuracy from a significant value. As an example, it raised the accuracy of the Extra Tree ensemble from 70.09% to 80.00%. Furthermore, Recursive Feature Elimination and the Feature Selection by Model methods provide better results compared to the chi-square test with a chi statistic greater than 2.0. To summarise the results, the best performing ensemble learner for four major emotions classification is the Extra Tree Classifier with the selected features listed in Table 6. These features are selected using the Model based feature selection approach by providing the Extra Tree Classifier as the feature selector.

According to the results presented in Table 6, most of the features from the TBF method got chosen as optimal features for emotion recognition. However, compared to the TFB method based features, EMD and HRV based features show less capability for emotion recognition. Most of the HRV features that were selected in the analysis contain statistical features of R–R interval variations. These features are quite similar to with-in beat analysis based features introduced in the literature that also have good capability (nearly one-third of them got selected). The only difference is that, compared to outer beat interval based statistical features used in HRV analysis, WIB analysis computes statistical features of the inner beat intervals of the ECG signal. Moreover, this further supports the fact that raw ECG patterns based features are the most efficient features for emotion recognition compared to different analysis based features.

Table 7 illustrate the results gathered from training different models with adopted emotions. Model A* is the principal model of this study and it has the ability to recognize four major emotions in the discrete emotion model with up to 80% accuracy. As mentioned, the experiment procedure also collected some additional emotions to prove the effectiveness of ensemble learning, and also for benchmarking aspects. Those models and their capabilities are also listed in the table, and the following paragraphs will compare those classification accuracies with the literature. Additionally, the table also depicts the individual gains of the Ten Frequency Band (TFB) analysis method introduced in this study. Even though those features do not comprise a direct physiological aspects of human emotions, those features tend to have a better performance compared to others. Therefore, it should be noted that more investigations should be conducted to evaluate the physiological aspects of those features.

As Table 7 shows, there is a significant accuracy improvement due to combining selected feature extraction methods with an ensemble learning process. The accuracy for identifying four major emotions is 80.00%, and this value is a significantly better result compared to the literature.

The method developed by Kim and André [8] combined four different sensors for detecting four primal emotions in the emotional spectrum and came up with an accuracy of 70%. However, the findings in this study provide insight into using a single sensor for developing the same classifier with an accuracy of 80.00%. Another investigation that was conducted to develop an emotion classification model by a neural network by Yoo et al. [28] achieved a recognition ability of 80% for identifying the four emotion quadrants using ECG and skin resistance. They have considered six subjects for their investigation, and the bio-signals were captured at different times of the day for a week. However, the classifier still presented in this investigation includes ECG patterns from 22 different subjects with a slightly lower accuracy of 78.12%. Furthermore, this study holds higher emotion recognition accuracy compared to the investigation by Maaoui et al. [51]. In their research, they have developed an emotion classifier to identify amusement, contentment, disgust, fear, neutral, and sadness from five biosensors. The accuracy of the developed model was 46.5%, and the method proposed in this investigation can still identify six emotions with up to 75.11% accuracy. Furthermore, the accuracy gained from this investigation outperforms several other studies that investigated multi-sensor based emotion recognition methods [52,53].

In another study, Murugappan et al. [34] developed an emotion recognition model for detecting five emotions (disgust, sad, joy, fear, neutral) which had an accuracy of 66.48%. However, they had 20 subjects for the experiment and some of the features that they considered include wavelet transformation based features. However, the method developed in this investigation has higher accuracy of 77.25% with a larger amount of subject space. The emotion recognition method developed utilizing with-in beat based features by Rattanyu and Mizukawa [9] had an emotion recognition accuracy of 61.44% for detecting six emotions, namely: anger; fear; sadness; joy; neutral; and digest. They had a smaller subject space compared to this study and the only difference between their model and the model C produced in this investigation is the digest emotion. The digest emotion falls into the same emotion quadrant of sadness, and, on the other hand, the pleasure emotion is in a different quadrant of the emotion model. The model developed in this study that replaced the digest emotion by pleasure had a recognition capability of 75.11% involving 22 subjects. Examining recent studies, the emotion recognition model developed by Guo et al. [54] has the capability to recognize anger, sadness, fear, joy and relax with up to 56.9% accuracy. In their investigation, they have used HRV analysis for the feature extraction and SVM was used as the machine learning model. By definition, the relaxed emotion in their study can be seen as the pleasure emotion adopted in this study, and, by considering that, their developed model has similarities to the model B developed in this study. Nevertheless, the model developed in this research outperforms their model by a 20% accuracy gain. Furthermore, the accuracy gained from combining all features from different domains has higher accuracies compared to emotion recognition studies that investigated the use of EMD based feature extraction methods [7,10].

Considering everything, it is apparent that the results obtained in this study outperform most of the methods mentioned in the literature. The next section describes additional perspectives of the proposed methodology such as emotion data collection methods, experiment procedures and accuracy gains of the TFB method introduced.

### 4.3. Results Overview

Table 8 compares the final results with the models developed in the literature. According to the data shown in the table, the combined analysis outperforms all ECG signal based emotion recognition models and a majority of models that use multiple biosensors for recognising emotions.

Compared to emotion recognition models that employ multiple biosensors, the frequency spectrum analysis technique introduced in this study has an accuracy enhancement of up to 24.13%. Furthermore, in contrast with the best performing ECG based emotion recognition model in the literature [34], the introduced ensemble model has an accuracy gain of 6.38%. Therefore, considering the accuracy improvement of the TFB method, it is apparent that the TFB method itself is an optimal method for emotion recognition using ECG signals.

Considering the combined analysis results, combining other broadly used techniques with the TFB method introduced has improved the prediction accuracy from a significant value. For instance, after incorporating other analysis based features with the TFB based model, the accuracy of the six emotion recognition model has improved by 4.48% (see Table 7). Furthermore, compared to the best ECG based emotion recognition model mentioned in the literature, the model developed by combining all four methods has improved the accuracy by 10.77%. Additionally, in contrast to the best performing multiple biosensor based emotion recognition model [28], the combined analysis based model has a similar accuracy with a significantly larger subject space.

As shown in Table 8, studies in the literature have used different methods to elicit emotions in their experimental procedure. The majority of them have used audio or video based methods to extract emotions, and most of them have been able to achieve high emotion recognition accuracy the same as in this study, which used video clips as the emotion elicitation method. It should be noted that, unlike picture based methods, these types of arrangements should use a sophisticated procedure to filter the emotion elicited climaxes from ECG signal space. Moreover, these videos should be picked carefully with the help of domain experts. Therefore, it is safe to say that the emotion-related data filtering protocol followed in this investigation is a reasonably fair approach for obtaining ECG signal based emotion climaxes recorded in the data (signal space). Regarding the number of subjects involved in an experiment, most of the single biosensor based studies have adopted a reasonably higher number of subjects, whereas multiple biosensor based studies have conducted experiments on a smaller number of subjects. However, as mentioned, the number of subjects involved in an experiment has a direct impact on the accuracy of the model. Therefore, the subject count in this analysis, which is 22, is a fairly higher value compared to other studies, and the model produced in this research still outperforms most of them.

Collectively, the ensemble learning method based model developed in this study holds a higher capability compared to other studies. Firstly, the combined analysis model comprises ECG emotional data from 22 subjects within the age range of 22–26. Secondly, the developed model is able to identify the emotion of a person from a 20 s ECG wave. Thirdly, the model uses a single biosensor for recognising emotions.

### 4.4. Computational Requirement Analysis

Table 9 illustrates the computational times and time and space complexities of the selected algorithms (*N* indicates the number of samples in the signal). According to the table, the EMD method takes 95.97% of the feature extraction time in the combined analysis.

Furthermore, comparing the additional space complexity added by the employed techniques, most of them used O(N) additional space while computing. In addition, feature extraction methods considered compute time domain and frequency domain features, and the computation time complexity of majority of methods has O(NlogN). Since the machine learning model takes a 20 s ECG wave, the combined computation time (0.23 s) will not affect the real-time nature of the system. Despite that, this will raise the model prediction accuracy from a significant amount.

Other than considering computational requirements for the feature extraction methods, while implementing real-life devices, several aspects related to model prediction complexity and the transmission time should be also concerned. In general, the prediction complexity of an Ensemble tree based classifier is $O(f_{n}\times n_{tree})$, where $f_{n}$ is the number of features and $n_{tree}$ is the number of trees in the ensemble. Furthermore, the transmission time of the signal solely depends on the method itself (i.e., is wired or wireless). The method used in this study, which is wired communication, might not be efficient in real scenarios. However, there has been an extensive amount of research going on in the domain of wearable computing that can be adopted to develop real-life applications [55,56].

## 5. Conclusions

The initial objective of this research was to evaluate the capability of ensemble learners for human emotion classification which needed an improved classification accuracy. According to the results presented, the combined features and the selected ensemble learners provide better performance compared to single learner models presented in the literature. Furthermore, the results presented in the feature selection based machine learning process proves that feature selection is a worthy step even for ensemble learners that rely on diversity.

Even though this study is not a review on ECG based emotion recognition, the results overview section provides an extensive review on ECG based feature extraction methods, emotion elicitation methods, experiment procedures and the evolution of ECG based human emotion recognition.

Findings from this research make several contributions to the current literature. Firstly, this research introduces the TFB analysis, which is a simple but efficient way for ECG based feature extraction, and the individual method has 6.38% accuracy gain compared to the best performing model in the literature. Even though the selected method does not have a physiological implication, the extracted features tend to have better capability in terms of signal based features. Secondly, findings from this research confirm that the ECG signal processing is an efficient method for bio-signal based emotion recognition. Furthermore, feature selection results of this analysis provide insight into the capability of the raw ECG signal pattern based features compared to different analysis based features. As the final contribution, this research provides empirical evidence on whether the feature selection prior to ensemble learning is an appropriate step or not. Taken together, models derived from selected features outperform all ECG signal based emotion recognition models mentioned in the literature with a classification accuracy gain of up to 28.61%.

## 6. Future Work

Further research needs to be done on emotion recognition for different age ranges. In addition, more research is also required to explore the capability of neural network based emotion recognition because neural networks features are engineered by themselves compared to traditional machine learning models which require pre-extracted features.

As mentioned, emotional intelligence can be applied to various situations where the interaction between the human and the machine (maybe a computer or a smartphone) needed to be improved and personalized. The analysis conducted in this study is based on a wired wearable device that has the ability to transfer data at a higher rate. However, this wired setup might not be feasible for real situations, and users might feel uncomfortable wearing these kinds of devices. However, recently, research has found ways of transmitting ECG data wirelessly by wearable devices [55,56]. Some of these designs can even be integrated into clothes themselves by using techniques such as capacitively coupled ECG [36,56,57]. Therefore, given that the computational complexity is low, as a real-life application, this model can be used even in a smartphone device.

## Figures and Tables

**Table 1:** Videos and subjective results.

Name	Target Emotion	Duration (minutes)	Hits	Misses
A scene from the Mr. Bean (1997) movie	Joy	5	25	0
A TV commercial about a pet	Sad	3.5	23	2
A 4K-HD video of space and landscape	Pleasure	5	18	2
A man beating a women in the streets (a viral video)	Anger	2	15	10
A movie seance from Mama (2013)	Fear	10	25	0
A black screen	Neutral	5	15	10

**Table 2:** HRV based features.

Type	Features
Time	*sdnn, mn_nn, rmssd, m_nn, nn50, pnn50*
Frequency	*hf, hfnu, lf, lf_hf, lfnu, total_power, vlf*
Geometric	*sd1, sd2*

**Table 3:** With-in beat features.

Interval	Features
PR	*min_pr, max_pr, sd_pr, mean_pr, median_pr*
ST	*min_st, max_st, sd_st, mean_st, median_st*
QRS	*min_qrs, max_qrs, sd_qrs, mean_qrs, median_qrs*

**Table 4:** Model evaluation results.

Classifier	Optimal Parameters	Accuracy
Random Forest	max_features: 2, n_estimators: 81, max_depth: 11	65.28%(5.08%)
Extra Tree Classifier	max_features: 6, n_estimators: 71, max_depth: 41	70.09%(3.34%)
Gradient Boost Classifier	n_estimators: 81, loss: deviance, learning_rate: 0.2	66.04%(4.07%)
ADABoost Classifier with SVM	n_estimators: 1, base_estimator: SVM(C=1.0, degree=3, gamma=’auto’, kernel=’rbf’)	41.25%(2.46%)
ADABoost Classifier with Decision Tree	n_estimators: 40, learning_rate: 1.0, algorithm: ’SAMME’	43.81%(4.19%)
ADABoost Classifier with Naive Bayes	n_estimators: 12, learning_rate: 1.3, algorithm: ’SAMME’	40.26%(5.35%)

**max_features:** maximum number of features considered while splitting, **n_estimators:** number of models (learners) in the ensemble, **max_depth:** maximum depth of a tree in ensemble, **base_estimator:** the estimator which is used to build the ensemble, **algorithm:** SAMME discrete boosting algorithm, **degree:** degree of the kernel, **kernel:** kernel type, **C:** penalty parameter.

**Table 5:** Ensemble methods with feature selection.

Method	RF	ETC	GB
RFE(number of features: 10)	64.34% (3.65%)	73.03% (3.35%)	68.97% (3.40%)
RFE(number of features: 15)	67.93% (2.64%)	66.13% (3.19%)	67.90% (4.47%)
RFE(number of features: 20)	66.92% (3.71%)	73.91% (2.61%)	69.21% (2.23%)
RFE(number of features: 25)	68.77% (3.34%)	74.00% (3.28%)	72.66% (3.36%)
RFE(number of features: 30)	65.60% (3.37%)	72.12% (3.44%)	65.11% (2.46%)
RFE(number of features: 35)	66.99% (5.12%)	73.00% (2.22%)	64.06% (4.56%)
RFE(number of features: 40)	63.71% (4.43%)	71.96% (3.87%)	61.12% (3.33%)
Chi-Squared statistic(>0.1)	67.51% (4.38%)	76.30% (3.84%)	65.06% (3.18%)
Chi-Squared statistic(>0.5)	59.69% (2.82%)	72.79% (3.23%)	64.77% (3.77%)
Chi-Squared statistic(>1.0)	63.47% (3.48%)	70.19% (2.55%)	63.66% (3.36%)
Chi-Squared statistic(>2.0)	52.80% (3.77%)	54.19% (2.34%)	54.44% (3.67%)
P-Test value (>0.1)	67.22% (4.33%)	76.22% (2.25%)	67.16% (3.47%)
P-Test value (>0.5)	67.76% (3.68%)	75.19% (4.04%)	68.00% (2.34%)
P-Test value (>0.8)	65.49% (3.36%)	70.21% (2.43%)	69.99% (3.37%)
Model based(model: RT)	75.35% (4.18%)	76.14% (4.04%)	71.62%(3.84%)
Model based(model: ETC)	79.23% (3.53%)	80.00% (4.27%)	71.40% (4.12%)
Model based(model: NB)	72.21% (3.87%)	77.13% (2.23%)	70.12%(4.22%)

**RF:** Random Forest, **ETC:** Extra Tree Classifier, **GB:** Gradient Boost Classifier, **RFE:** Recursive Feature Elimination, **NB:** Naive Bayes.

**Table 6:** Feature selection results.

Method	N	D	Features
EMD	$\frac{6}{24}$	T	*spec_p_1 ,* ***spec_p_2*** *, spec_p_3,* ***spec_p_4*** *,spec_p_1, spec_p_6, ins_p_1, ins_p_2, ins_p_3, ins_p_4, ins_p_5, ins_p_6*
		F	*spec_pf_1,* ***mean_if_1*** *,* ***spec_pf_2*** *, mean_if_2, spec_pf_3, mean_if_3,* ***spec_pf_4*** *, mean_if_4, spec_pf_5,* ***mean_if_5*** *, spec_pf_6, mean_if_6*
HRV	$\frac{3}{14}$	T	***sdnn*** *,* ***mn_nn*** *, pnn50,* ***m_nn*** *, rmssd, nn50*
		F	*lf, ffnu, lf_hf, total_power, hfnu, vlf*
		G	*sd1, sd2*
WIB	$\frac{6}{15}$	T	***median_pr*** *, mean_pr,* ***max_pr*** *,* ***sd_pr*** *, min_pr, median_qrs,* ***mean_qrs*** *,* ***max_qrs*** *,* ***min_qrs*** *, sd_qrs, median_st, max_st, min_st, mean_st, sd_st*
FFB	$\frac{6}{10}$	F	*band_1,* ***band_2*** *,* ***band_3*** *, band_4,* ***band_5*** *,* ***band_6*** *,* ***band_7*** *, band_8, band_9,* ***band_10***

**D** = Domain, **T** = Time Domain, **F** = Frequency Domain; **G** = Geometric; **N** = selected features as a fraction of total features extracted from the method.

**Table 7:** Benchmark models.

Model	Emotions	Accuracy(TFB)	Accuracy
A*	anger, sadness, joy, pleasure	75.94%(4.11%)	80.00%(4.27%)
B	anger, sadness, joy, pleasure, fear	72.86%(3.47%)	77.25%(3.14%)
C	four emotion quadrants	72.13%(3.26%)	78.12%(4.32%)
D	anger, sadness, joy, pleasure, fear, neutral	70.63%(3.77%)	75.11%(3.77%)

**Table 8:** Comparison with the literature.

Study	Adopted Emotions	E-EM	MS	N	Acc	Gain(%)	
						TFB	Com
Kim et al. (2004) [52]	sadness, anger, stress, surprise	A	✓	124	61.8%	+14.14	+18.20
Yoo et al. (2005) [28]	sadness, calm pleasure, interesting pleasure, fear (four quadrants)	V	✓	6	80%	-7.87	-0.21
Rigas et al. (2007) [53]	joy, disgust, fear	P	✓	9	62.7%		
Kim and André (2008) [8]	anger, sadness, pleasure, joy	A	✓	3	70%	+5.94	+10.00
Maaoui et al. (2010) [51]	amusement, contentment, disgust, fear, sadness, neutral	I	✓	10	46.5%	+24.13	+28.61
Rattanyu and Mizukawa (2011) [9]	anger, fear, sadness, joy, digest, neutral	P	✗	12	61.44%	+9.19	+8.67
Jeritta et al. (2012) [7]	neutral, happiness, sadness, fear, surprise, disgust	V	✗	15	59.78%	+10.85	+15.33
Murugappan et al. (2013) [34]	digest, sadness, fear, joy, neutral	V	✗	20	66.48%	+6.38	+10.77
Jerritta et al. (2014) [10]	neutral, happiness, sadness, fear, surprise, disgust	V	✗	30	54%	+16.63	+21.11
Guo et al. (2016) [54]	sadness, angry, fear, happy, relaxed	V	✗	25	56.9%	+15.96	+20.35

**E-EM**= emotion elicited method, **N**= number of subjects, **MS**= Multiple Sensors including ECG sensor, **ECG-FE**= ECG signal Feature Extraction method, **IM+**= Improvement after combining. **A:** Audio, **I:** Images, **V:** Video and **Acc:** Accuracy.

**Table 9:** Computational requirements.

Method	Computation Time(s)	Space Complexity	Time Complexity
HRV	0.0016 (0.69%)	O(N)	O(NlogN)
EMD	0.2216 (95.97%)	O(N)	O(NlogN)
WIB	0.0004 (0.17%)	O(1)	O(N)
TFB	0.0023 (0.99%)	O(N)	O(NlogN)
Combined	0.2309	O(N)	O(NlogN)

## References

[1] Cowie R., Douglas-Cowie E., Taylor J., Ioannou S., Wallace M., Kollias S. An Intelligent System for Facial Emotion Recognition. Proceedings of the 2005 IEEE International Conference on Multimedia and Expo.

[2] Tu C.-T., Lien J.J.J. (2010). Automatic Location of Facial Feature Points and Synthesis of Facial Sketches Using Direct Combined Model. IEEE Trans. Syst. Man Cybern. Part B (Cybern.).

[3] Lee C.M., Narayanan S. (2005). Toward detecting emotions in spoken dialogs. IEEE Trans. Speech Audio Process..

[4] Cook N., Fujisawa T., Takami K. (2006). Evaluation of the affective valence of speech using pitch substructure. IEEE Trans. Audio Speech Lang. Process..

[5] Parsons T.D., Reinebold J.L. (2012). Adaptive virtual environments for neuropsychological assessment in serious games. IEEE Trans. Consum. Electron..

[6] Tokuno S., Tsumatori G., Shono S., Takei E., Yamamoto T., Suzuki G., Mituyoshi S., Shimura M. Usage of emotion recognition in military health care. Proceedings of the 2011 Defense Science Research Conference and Expo (DSR).

[7] Jerritta S., Murugappan M., Wan K., Yaacob S. Emotion recognition from electrocardiogram signals using Hilbert Huang Transform. Proceedings of the 2012 IEEE Conference on Sustainable Utilization and Development in Engineering and Technology, STUDENT 2012 - Conference Booklet.

[8] Kim J., André E. (2008). Emotion recognition based on physiological changes in music listening. IEEE Trans. Pattern Anal. Mach. Intell..

[9] Rattanyu K., Mizukawa M. (2011). Emotion recognition based on ecg signals for service robots in the intelligent space during daily life. J. Adv. Comput. Intell. Intell. Inf..

[10] Jerritta S., Murugappan M., Wan K., Yaacob S. (2014). Electrocardiogram-based emotion recognition system using empirical mode decomposition and discrete Fourier transform. Expert Syst..

[11] Bexton R.S., Vallin H.O., Camm A.J. (1986). Diurnal variation of the QT interval–influence of the autonomic nervous system. Br. Heart J..

[12] Ayata D., Yaslan Y., Kamasak M. Emotion recognition via random forest and galvanic skin response: Comparison of time based feature sets, window sizes and wavelet approaches. Proceedings of the 2016 Medical Technologies National Congress (TIPTEKNO).

[13] Opitz D.W. (1999). Feature Selection for Ensembles. Proceedings of the Sixteenth National Conference on Artificial Intelligence and the Eleventh Innovative Applications of Artificial Intelligence Conference Innovative Applications of Artificial Intelligence.

[14] Khoshgoftaar T.M., Gao K., Napolitano A. Improving software quality estimation by combining feature selection strategies with sampled ensemble learning. Proceedings of the 2014 IEEE 15th International Conference on Information Reuse and Integration (IEEE IRI 2014).

[15] Maglaras L.A., Jiang J., Cruz T.J. (2016). Combining ensemble methods and social network metrics for improving accuracy of OCSVM on intrusion detection in SCADA systems. J. Inf. Secur. Appl..

[16] Mahdavi-Shahri A., Houshmand M., Yaghoobi M., Jalali M. Applying an ensemble learning method for improving multi-label classification performance. Proceedings of the 2016 2nd International Conference of Signal Processing and Intelligent Systems (ICSPIS).

[17] Hosseini M.P., Hajisami A., Pompili D. Real-Time Epileptic Seizure Detection from EEG Signals via Random Subspace Ensemble Learning. Proceedings of the 2016 IEEE International Conference on Autonomic Computing (ICAC).

[18] Jin L.p., Dong J. (2016). Ensemble Deep Learning for Biomedical Time Series Classification. Comput. Intell. Neurosci..

[19] Pujari P., Gupta J.B. (2012). Improving Classification Accuracy by Using Feature Selection and Ensemble Model. Int. J. Soft Comput. Eng..

[20] Gao K., Khoshgoftaar T., Wald R. Combining feature selection and ensemble learning for software quality estimation. Proceedings of the 27th International Florida Artificial Intelligence Research Society Conference.

[21] Christopher B., Narayan D. (2015). Biofeedback: A Player’s Anxiety as Input into a Video Game Environment. Proceedings of the AASRI International Conference on Industrial Electronics and Applications (2015).

[22] Pejman M.b., Sebastian L., Emma F. Understanding the Contribution of Biometrics to Games User Research. Proceedings of the 2011 DiGRA International Conference: Think Design Play.

[23] Katsis C., Katertsidis N., Ganiatsas G., Fotiadis D. (2008). Toward Emotion Recognition in Car-Racing Drivers: A Biosignal Processing Approach. IEEE Trans. Syst. Man Cybern. Part A Syst. Humans.

[24] Eyben F., Wöllmer M., Poitschke T., Schuller B., Blaschke C., Färber B., Nguyen-Thien N. (2010). Emotion on the Road—Necessity, Acceptance, and Feasibility of Affective Computing in the Car. Adv. Hum.-Comput. Interact..

[25] Lisetti C., Nasoz F., LeRouge C., Ozyer O., Alvarez K. (2003). Developing multimodal intelligent affective interfaces for tele-home health care. Int. J. Hum.-Comput. Stud..

[26] Olfson M., Gilbert T., Weissman M., Blacklow R.S., Broadhead W. (1995). Recognition of emotional distress in physically healthy primary care patients who perceive poor physical health. Gen. Hosp. Psychiatry.

[27] Paithane A.N., Bormane D.S., Tahawade R.S.C.O.E. (2014). Human Emotion Recognition using Electrocardiogram Signals. Int. J. Recent Innov. Trends Comput. Commun..

[28] Yoo S.K., Lee C.K., Park Y.J., Kim N.H., Lee B.C., Jeong K.S. (2005). Neural Network Based Emotion Estimation Using Heart Rate Variability and Skin Resistance.

[29] Ayata D., Yaslan Y., Kamaşak M. (2017). Emotion Recognition via Galvanic Skin Response: Comparison of Machine Learning Algorithms and Feature Extraction Methods. Istanb. Univ. J. Electr. Electron. Eng..

[30] Nasoz F., Alvarez K., Lisetti C.L., Finkelstein N. (2004). Emotion recognition from physiological signals using wireless sensors for presence technologies. Cogn. Technol. Work.

[31] Gouizi K., Bereksi Reguig F., Maaoui C. (2011). Emotion recognition from physiological signals. J. Med Eng. Technol..

[32] Jerritta S., Murugappan M., Nagarajan R., Wan K. Physiological signals based human emotion Recognition: A review. Proceedings of the 2011 IEEE 7th International Colloquium on Signal Processing and its Applications.

[33] Egger M., Ley M. (2019). Emotion Recognition from Physiological Signal Analysis: A Review. Electron. Notes Theor. Comput. Sci..

[34] Murugappan M., Murugappan S., Zheng B.S. (2013). Frequency Band Analysis of Electrocardiogram (ECG) Signals for Human Emotional State Classification Using Discrete Wavelet Transform (DWT). J. Phy. Ther. Sci..

[35] Xu Y., Liu G., Hao M., Wen W., Huang X. (2010). Analysis of affective ECG signals toward emotion recognition. J. Electron. (China).

[36] Nemati E., Deen M.J., Mondal T. (2012). A Wireless Wearable ECG Sensor for Long-Term Applications Studying heart failure and adverse outcomes in paediatric heart disorders View project nanomaterials View project. IEEE Commun. Mag..

[37] Heart and Brain SpikerShield Bundle. https://backyardbrains.com/products/heartandbrainspikershieldbundle.

[38] Ferdinando H., Seppanen T., Alasaarela E. Comparing features from ECG pattern and HRV analysis for emotion recognition system. Proceedings of the 2016 IEEE Conference on Computational Intelligence in Bioinformatics and Computational Biology (CIBCB).

[39] Bernardo A.F.B., Vanderlei L.C.M., Garner D.M. (2014). HRV Analysis: A Clinical and Diagnostic Tool in Chronic Obstructive Pulmonary Disease. Int. Sch. Res. Not..

[40] Izard C.E., Libero D.Z., Putnam P., Haynes O.M. (1993). Stability of emotion experiences and their relations to traits of personality. J. Personal. Soc. Psychol..

[41] Huang N.E., Shen Z., Long S.R., Wu M.C., Shih H.H., Zheng Q., Yen N.C., Tung C.C., Liu H.H. (1998). The empirical mode decomposition and the Hilbert spectrum for nonlinear and non-stationary time series analysis. Proc. R. Soc. London. Ser. A: Math. Phys. Eng. Sci..

[42] Singh Rupal H., Mohanty S., Kishor N., Singh D. Comparison of Empirical Mode Decomposition and Wavelet Transform for Power Quality Assessment in FPGA. Proceedings of the 2018 IEEE International Conference on Power Electronics, Drives and Energy Systems (PEDES).

[43] Agrafioti F., Hatzinakos D., Anderson A.K. (2012). ECG Pattern Analysis for Emotion Detection. IEEE Trans. Affect. Comput..

[44] Emotion Recognition based on Heart Rate and Skin Conductance. Proceedings of the 2nd International Conference on Physiological Computing Systems, SCITEPRESS - Science and and Technology Publications.

[45] Russell J.A. (1979). Affective space is bipolar. J. Personal. Soc. Psychol..

[46] Gross J.J., Levenson R.W. (1995). Emotion elicitation using films. Cogn. Emot..

[47] Ashley E.A., Niebauer J. (2004). Conquering the ECG.

[48] Welch P. (1967). The use of fast Fourier transform for the estimation of power spectra: A method based on time averaging over short, modified periodograms. IEEE Trans. Audio Electroacoust..

[49] Jones E., Oliphant T., Peterson P. SciPy: Open source scientific tools for Python. http://www.scipy.org/.

[50] Pedregosa F., Varoquaux G., Gramfort A., Michel V., Thirion B., Grisel O., Blondel M., Prettenhofer P., Weiss R., Dubourg V. (2011). Scikit-learn: Machine Learning in Python. J. Mach. Learn. Res..

[51] Maaoui C., Pruski A. (2010). Emotion Recognition through Physiological Signals for Human-Machine Communication. Cutting Edge Robotics 2010.

[52] Kim K.H., Bang S.W., Kim S.R. (2004). Emotion recognition system using short-term monitoring of physiological signals. Med. Biol. Eng. Comput..

[53] Rigas G., Katsis C.D., Ganiatsas G., Fotiadis D.I. (2007). A User Independent, Biosignal Based, Emotion Recognition Method. User Modeling 2007.

[54] Guo H.W., Huang Y.S., Lin C.H., Chien J.C., Haraikawa K., Shieh J.S. Heart Rate Variability Signal Features for Emotion Recognition by Using Principal Component Analysis and Support Vectors Machine. Proceedings of the 2016 IEEE 16th International Conference on Bioinformatics and Bioengineering (BIBE).

[55] Park C., Chou P.H., Bai Y., Matthews R., Hibbs A. An ultra-wearable, wireless, low power ECG monitoring system. Proceedings of the 2006 IEEE Biomedical Circuits and Systems Conference.

[56] Van Den Broek E.L., Schut M.H., Westerink J.H.D.M., Tuinenbreijer K. (2009). Unobtrusive Sensing of Emotions (USE). J. Ambient Intell. Smart Environ..

[57] Yama Y., Ueno A., Uchikawa Y. Development of a Wireless Capacitive Sensor for Ambulatory ECG Monitoring over Clothes. Proceedings of the 2007 29th Annual International Conference of the IEEE Engineering in Medicine and Biology Society.

